# Interconnected Reservoirs: Virulence & Biofilm Traits of ESBL-*Klebsiella pneumoniae* in Municipal Wastewater & Agricultural Systems

**DOI:** 10.3390/microorganisms14071435

**Published:** 2026-06-30

**Authors:** Mabel Kamweli Aworh, Courtney W. Reggans, Monica S. Sellars, Jordan C. Deutschlander, Isaiah J. Taylor, Deepa Gopal Struble, Katrina L. Edwards, Lyndy Harden, Rhonda Locklear, Kristen Delaney Nguyen

**Affiliations:** 1 Department of Biological and Forensic Sciences, Fayetteville State University, Fayetteville, NC 28301, USA; creggans@broncos.uncfsu.edu (C.W.R.); msellars2@broncos.uncfsu.edu (M.S.S.); jdeutschlander@broncos.uncfsu.edu (J.C.D.); itaylor9@broncos.uncfsu.edu (I.J.T.); dstruble@broncos.uncfsu.edu (D.G.S.); kedwards7@broncos.uncfsu.edu (K.L.E.); kdelaney@uncfsu.edu (K.D.N.); 2 Department of Population Health and Pathobiology, College of Veterinary Medicine, North Carolina State University, Raleigh, NC 27607, USA; lbharden@ncsu.edu; 3 Fayetteville Public Works Commission, Fayetteville, NC 28301, USA; rhonda.locklear@faypwc.com

**Keywords:** wastewater surveillance, biofilm formation, virulence genes, one health, phylogenomics, wastewater treatment plants, environmental dissemination

## Abstract

Extended-spectrum β-lactamase-producing *Klebsiella pneumoniae* (ESBL-KP) is an important antimicrobial-resistant pathogen, and wastewater may serve as a reservoir for its persistence and dissemination. This study investigated the virulence-associated genes, biofilm-forming capacity and genomic relatedness of ESBL-KP isolates recovered from wastewater and livestock farm environments in southeastern North Carolina. A cross-sectional study was conducted between May and September 2025 at two wastewater treatment plants (WWTPs) and two livestock farms. ESBL-KP isolates recovered from wastewater, animal feces, and water samples were characterized using PCR, whole-genome sequencing and crystal violet biofilm assays. Genomic relatedness was assessed using phylogenomic analysis. Data were analyzed using descriptive statistics and Fisher’s exact test. ESBL-KP was detected in 15.4% (*n* = 69/449) of samples, with the highest prevalence observed in WWTPs (75.4%, *n* = 52) followed by poultry farms (21.7%, *n* = 15). The most frequent virulence genes were *mrkD* (30/69), *entB* (26/69), *K2* (21/69), and *rmpA* (21/69). Significant variation in gene distribution by sample type was observed for *mrkD* (*p* = 0.0013) and *entB* (*p* = 0.0011). Biofilm formation varied by sample type, with strong biofilm predominating in influent (*n* = 20) and sludge (*n* = 8), although no significant differences were detected across sample types (*p* = 0.357). Phylogenetic analysis revealed that one poultry farm isolate was clonally related to wastewater isolates, differing by 1–3 single nucleotide polymorphisms (SNPs) and sharing the virulence genes *mrkA*, *iutA*, and *fimH*. Overall, environmental ESBL-KP isolates exhibited widespread virulence potential and robust biofilm-forming capacity, while phylogenetic evidence demonstrated clonal relatedness between poultry farm and wastewater isolates and sharing *mrkA*, *iutA*, and *fimH* virulence genes. These findings highlight wastewater and agricultural systems as genetically related reservoirs for clinically relevant ESBL-KP strains and underscore the need for strengthened One Health-based surveillance to monitor and mitigate their environmental dissemination.

## 1. Introduction

*Klebsiella pneumoniae* is a Gram-negative, encapsulated bacterium belonging to the Enterobacteriaceae family and is recognized as one of the most clinically significant opportunistic pathogens worldwide [1,2,3]. Although *K. pneumoniae* naturally exists in environmental reservoirs such as soil, water, wastewater, plants, and animal-associated environments, it is also responsible for a wide range of human infections [1,4,5]. *K. pneumoniae* is a major ESKAPE pathogen associated with severe infections, including pneumonia, urinary tract infections, septicemia, liver abscesses, and a wide range of healthcare-associated infections [6,7]. The growing concern surrounding *K. pneumoniae* stems from its increasing antimicrobial resistance (AMR), virulence-associated characteristics, and ability to persist in environmental and clinical settings through biofilm formation [1,2,6]. These characteristics have elevated the organism into a major global public health threat and a significant focus of microbiological and epidemiological research [1,6].

Globally, AMR *K. pneumoniae* infections have increased dramatically over the last two decades [8,9]. The World Health Organization (WHO) has identified carbapenem-resistant and extended-spectrum beta-lactamase (ESBL)-producing *K. pneumoniae* as priority pathogens requiring urgent research and development of new antimicrobial therapies [10]. Several studies have demonstrated that environmental reservoirs play a substantial role in the persistence and dissemination of these organisms [9,11]. Wastewater systems, agricultural runoff, livestock environments, and contaminated surface waters have all been identified as important ecological niches where *K. pneumoniae* can survive, exchange genetic material, and potentially spread virulence and resistance genes [1,12,13,14]. Environmental surveillance studies conducted in Asia, Europe, Africa, and South America have reported the recovery of *K. pneumoniae* strains carrying virulence genes and antimicrobial resistance determinants from municipal wastewater treatment plants and agricultural systems [6,8,9].

Environmental AMR surveillance is increasingly critical in the United States due to growing AMR threats and public-health preparedness needs [15]. Wastewater-based AMR surveillance has expanded rapidly as researchers recognize wastewater systems as major reservoirs and monitoring points for emerging pathogens [15,16,17]. Studies across the U.S. have detected ESBL-producing *K. pneumoniae* in wastewater treatment plants (WWTPs), sewage networks, agricultural runoff, and recreational waters [17,18]. These findings show that municipal wastewater collects bacteria from hospitals, households, livestock, and industry, making WWTPs critical sites where antibiotic-resistant and virulent strains can accumulate and persist [19].

*K. pneumoniae* carries multiple virulence genes—such as *mrkD*, *entB*, *iutA*, *allS*, and *kfu*—that enhance adhesion, biofilm formation, iron acquisition, metabolism, and overall pathogenicity [2,20]. These factors improve survival in both environmental and host settings and increase the organism’s ability to cause severe infection [20]. Another major factor contributing to the persistence of *K. pneumoniae* is biofilm formation [21,22]. Biofilms are surface-attached microbial communities that protect bacteria from antibiotics, disinfectants, UV exposure, and environmental stress [11,22,23]. In these structures, cells readily exchange resistance and virulence genes, accelerating the spread of multidrug-resistant strains [21,23]. Biofilm-producing *K. pneumoniae* is strongly linked to persistent infections and elevated resistance, making assessment of biofilm formation essential for understanding the organism’s environmental persistence and transmission potential [22,23,24].

The One Health framework highlights how human, animal, and environmental health are interconnected, with pathogens and AMR moving across these systems [8,13]. Wastewater and agricultural settings act as key convergence points where microbes from hospitals, households, livestock, and industry mix, creating opportunities for persistence, adaptation, and cross-ecosystem transmission [16,19].

Environmental surveillance data for virulence-associated *K. pneumoniae* in North Carolina remain limited, despite the state’s extensive agriculture and numerous wastewater facilities. While some studies have examined AMR in wastewater [25], few studies elsewhere have focused specifically on *K. pneumoniae* virulence genes or biofilm traits [2,11]. Within Fayetteville, North Carolina, limited published data are available regarding the molecular characteristics and biofilm-forming capabilities of environmental *K. pneumoniae* isolates. Fayetteville is a key site for environmental surveillance because of its wastewater systems, agricultural activity, growing population, and proximity to healthcare and military facilities. Monitoring these environments can reveal circulation patterns of virulent strains and strengthen regional public-health surveillance. These gaps highlight the need for localized data, especially as environmental reservoirs gain importance within the One Health framework.

Therefore, the purpose of this study was to investigate the molecular detection of virulence-associated genes and evaluate biofilm formation among *K. pneumoniae* isolates recovered from environmental sources in North Carolina, including municipal WWTPs, poultry farms, and cattle farms in Fayetteville.

## 2. Materials and Methods

### 2.1. Study Design and Site Selection

A cross-sectional study was conducted between May and September 2025 to investigate the prevalence of virulence-associated genes and biofilm formation among environmental *Klebsiella pneumoniae* isolates recovered from wastewater and agricultural sources in North Carolina. Sampling locations were selected to represent interconnected environmental reservoirs within the One Health framework, including municipal wastewater treatment plants (WWTPs), poultry farms, and cattle farms in and around Fayetteville, North Carolina. Two municipal WWTPs and two agricultural facilities consisting of poultry and cattle operations were included in the study. These sites were selected because wastewater systems and agricultural environments are recognized as important reservoirs for antimicrobial-resistant and virulent bacterial organisms.

### 2.2. Sample Collection

Environmental samples were collected aseptically from wastewater and agricultural sources. Wastewater samples (1 L) were obtained from influent and effluent collection points at municipal WWTPs using sterile sampling containers. Agricultural samples included cattle feces, poultry litter, soil, and water samples collected from farm environments. Samples were transported to the laboratory on ice and processed within two hours of collection to preserve bacterial viability.

A total of 449 environmental samples were collected during the study period. Each sample was assigned a unique identification number to ensure traceability throughout microbiological and molecular analyses. Standard biosafety and sterile handling procedures were followed during sample collection, transportation, and laboratory processing.

### 2.3. Bacterial Isolation and Identification

Environmental samples were cultured on selective and differential media to isolate Gram-negative enteric bacteria. Presumptive *K. pneumoniae* isolates were selected based on colony morphology and lactose fermentation characteristics. Pure isolates were obtained through repeated streak plating using MacConkey agar (Becton, Dickinson and company, Sparks, MD, USA) supplemented with 1 μg/mL cefotaxime (Sigma-Aldrich, St. Louis, MO, USA) and incubated at 37 °C for 18–24 h. Preliminary identification was performed using standard biochemical testing procedures, including Triple Sugar Iron (TSI) agar (Oxoid, Basingstoke, UK), Citrate agar (Oxoid, Basingstoke, UK), and Lysine Iron Agar (LIA) (Becton, Dickinson and company, Sparks, MD, USA), to confirm the identification of *Klebsiella* species.

Following initial screening on cefotaxime-supplemented MacConkey agar (Becton, Dickinson and company, Sparks, MD, USA), all presumptive ESBL-producing isolates were subjected to phenotypic confirmation using the CLSI-recommended combined disk diffusion method with cefotaxime and ceftazidime, each tested with and without clavulanic acid (Becton, Dickinson and company, Sparks, MD, USA). Isolates demonstrating a ≥5 mm increase in inhibition zone diameter in the presence of clavulanate were classified as ESBL producers. Quality control for ESBL detection included the use of *K. pneumoniae* ATCC 700603 as the ESBL-positive control and *Escherichia coli* ATCC 25922 as the ESBL-negative control. Confirmed isolates were preserved in 15% glycerol–tryptone soy broth and stored at −80 °C until molecular analysis was performed.

### 2.4. Detection of Virulence Genes

#### 2.4.1. DNA Extraction

Genomic DNA was extracted from overnight bacterial cultures using a boiling lysis preparation method adapted from Compain et al. [26]. Briefly, bacterial isolates stored in freezer stock cultures were streaked onto agar plates and incubated overnight at 37 °C. Individual colonies were selected using sterile 10 µL inoculating loops and suspended in 150 µL of sterile distilled water in sterile 1.5 mL microcentrifuge tubes. The bacterial suspensions were vortexed thoroughly to ensure homogenization and then heated at 100 °C for approximately 15 min to lyse bacterial cells and release genomic DNA. Following heat treatment, samples were centrifuged for 5 min to pellet cellular debris. The resulting supernatant containing crude DNA template was transferred and stored at refrigeration temperature until analysis.

#### 2.4.2. Polymerase Chain Reaction (PCR) Assays

Multiplex polymerase chain reaction (PCR) assays adapted from Compain et al. were performed to detect virulence-associated genes in environmental *K. pneumoniae* isolates [26]. We had two groups of multiplex PCR, A and B. The target genes for multiplex PCR assay A included *mrkD*, *entB*, *allS*, *iutA*, and *kfu*, which are associated with adhesion, siderophore production, iron acquisition, and virulence potential. Multiplex PCR assay B targeted *ybtS*, *rmpA*, *magA*, and *K2*, genes associated with siderophore production, hypermucoviscosity, and capsular serotypes linked to increased virulence. Primers utilized in this study were selected from previously published multiplex PCR protocols developed for *K. pneumoniae* virulence gene detection and are presented in Table 1 [26]. Primer working stocks were diluted to a final concentration of 10 µM prior to use. The master mixes were prepared on ice according to the virulence multiplex PCR protocol. Each 25 µL reaction contained molecular-grade water, DreamTaq PCR Master Mix (2X) (Thermo Scientific, Waltham, MA, USA), forward and reverse primers for each virulence target gene, and 1 µL of DNA template. Negative and positive controls were included in each PCR run to ensure assay accuracy and contamination control. PCR amplification was performed under the following thermal cycling parameters for PCR A and B: an initial denaturation step at 95 °C for 15 min, followed by 30 amplification cycles consisting of denaturation at 94 °C for 30 s, annealing at 60 °C for 90 s and extension at 72 °C for 60 s, and final extension at 72 °C for 10 min. All PCR experiments were performed on a SimpliAmpTM Thermal cycler (ThermoFisher Scientific, Waltham, MA, USA).

PCR products were analyzed using 2% agarose gel electrophoresis prepared with SYBR Safe DNA gel stain (Thermo Fisher Scientific, Carlsbad, CA, USA). Agarose gels were prepared by adding 5 µL of SYBR Safe stain per 50 mL of molten agarose according to laboratory protocol. Amplified PCR products and the Invitrogen TrackIt 1 Kb Plus DNA Ladder (Thermo Fisher Scientific, Vilnius, Lithuania) were loaded into gel wells and electrophoresed at 100 V for approximately 2 h. DNA fragments were visualized using the Invitrogen iBright FL1500 Imaging System (ThermoFisher Scientific, Waltham, MA, USA) under nucleic acid imaging settings. Bands were compared with the molecular weight ladder to determine the presence or absence of target virulence genes based on the expected amplicon sizes. Gel images were documented and interpreted manually. Isolates demonstrating visible bands at expected molecular weights were recorded as positive for the corresponding virulence gene, while isolates lacking visible amplification bands were considered negative.

### 2.5. DNA Extraction, Library Preparation, and Whole-Genome Sequencing

A subset of phenotypically confirmed generic ESBL-producing *K. pneumoniae* isolates was selected for whole-genome sequencing (WGS) based on source type and detected virulence gene profiles (*n* = 27). Genomic DNA was extracted using the DNeasy PowerLyzer Microbial Kit (Qiagen, Germantown, MD, USA) according to the manufacturer’s instructions. Briefly, bacterial colonies were harvested from culture plates and suspended in Power Bead Solution and SL Solution, followed by mechanical disruption using a Bead Mill 24 homogenizer (Omni, Kennesaw, GA, USA). After centrifugation, the supernatant underwent sequential purification steps using IRS and SB Solutions before being transferred onto spin columns. The columns were washed with CB Solution, and purified DNA was eluted in 50 μL of EB Solution. DNA concentration and purity were assessed using a NanoDrop spectrophotometer (ThermoFisher Scientific, Waltham, MA, USA).

Genomic libraries with an average insert size of approximately 350 bp were prepared using the Illumina DNA Prep Kit and Illumina DNA/RNA UD Indexes (Illumina, San Diego, CA, USA). Libraries were pooled and sequenced on the Illumina NextSeq 2000 platform using the NextSeq 1000/2000 P1 Reagent Kit (Illumina, San Diego, CA, USA) (600 cycles) to generate paired-end 250 bp reads.

Sequence quality assessment and downstream analyses were performed using the MicroRunQC workflow within the GalaxyTrakr platform [27]. Analyses included quality evaluation of raw paired-end FASTQ reads and de novo genome assembly. Assembly metrics such as genome size, number of contigs, N50 values, sequence type (ST), median insert size, and mean read length were generated for each isolate. Sequencing data met established quality thresholds, including minimum read depth, assembly completeness, and contamination screening.

Species identity was confirmed using genome-based analyses, including average nucleotide identity (ANI) and phylogenomic comparison with established *Klebsiella* reference genomes.

### 2.6. Bioinformatics and Phylogenetic Analysis

Raw sequencing reads were quality-filtered, trimmed, and assembled de novo using SPAdes v4.2.0 [28]. Genome annotation was performed with Prokka v1.14.0 to identify coding sequences and genomic features [29]. A pan-genome analysis was conducted using Panaroo v1.5.1 to identify the core genome and generate a core genome alignment [30]. Single nucleotide polymorphism (SNP) positions were extracted from the core genome alignment using SNP-sites v2.5.1 [31], and pairwise SNP distances were calculated using SNP-dists v0.8.2 [32]. A maximum-likelihood phylogenetic tree was constructed using RAxML v8.2.13 (RaxmlHPC) under the GTRGAMMA nucleotide substitution model with a parsimony starting tree and a random seed of 12345 [33]. The resulting phylogeny was used to assess the genetic relatedness, clustering, and potential clonality of isolates recovered from municipal wastewater and farm environments.

Sequencing data that passed quality control requirements, along with associated metadata, were deposited in the National Center for Biotechnology Information (NCBI) Pathogen Detection database under the BioProject accession PRJNA1092662, where individual isolates received Sequence Read Archive (SRA) and BioSample accession numbers. Phylogenetic trees and associated metadata were visualized using iTOL v7 [34]. Accession numbers for all sequenced isolates are provided in Supplementary Table S1.

### 2.7. Detection of Mobile Genetic Elements

Mobile genetic elements (MGEs) were identified using MobileElementFinder v1.0.3 (Center for Genomic Epidemiology, CGE) [35]. Draft genome assemblies were screened against the MobileElementFinder database v1.0.2 to detect insertion sequences, transposons, and other MGEs. Analyses were performed using the default detection thresholds of ≥90% nucleotide identity and ≥60% sequence coverage. The distribution of MGEs among isolates was subsequently compared in isolates from different sources.

### 2.8. Biofilm Formation Assay

#### 2.8.1. Inoculum Preparation

Bacterial isolates were first streaked onto Tryptone Soy agar (Becton, Dickinson and company, Sparks, MD, USA), plates using aseptic technique and incubated at 37 °C for 18–24 h until visible colonies formed. Individual colonies from overnight cultures were then suspended in sterile diluent and standardized using the 0.5 McFarland turbidity standard to ensure consistent bacterial concentration across all samples. Positive and negative control strains were included for comparison of biofilm-forming ability.

#### 2.8.2. Microtiter Plate Assay

The microtiter plate assay was performed using sterile 96-well polystyrene plates. Biofilm culture medium was prepared by combining 20 mL of 5× M9 medium with 80 mL distilled water, followed by the addition of casamino acids, MgSO_4_, CaCl_2_, thiamine, and glucose [36]. We pipetted 90 µL of broth medium into each well and 10 µL of standardized bacterial suspension in triplicate. Blank wells containing sterile diluent served as negative controls. A known strong biofilm-producing *K. pneumoniae* strain, previously confirmed by WGS, was used as the positive control. Plates were incubated overnight at 37 °C to allow biofilm formation.

#### 2.8.3. Quantification of Biofilm Formation

Following incubation, planktonic cells were removed by discarding the culture supernatant and gently blotting the microtiter plate onto absorbent paper. Wells were stained with 125 µL of 0.03% crystal violet solution for 15 min at room temperature using a digital microplate shaker (ThermoFisher Scientific, Waltham, MA, USA). Excess stain was removed, and wells were washed three times with sterile distilled water to eliminate unbound crystal violet. The bound crystal violet was solubilized with 125 µL of 100% ethanol, and biofilm biomass was quantified by measuring optical density at 570 nm (OD570) using a Tecan Sunrise absorbance microplate reader (Tecan, Mannedorf, Switzerland). Each isolate was assayed in triplicate, and the mean OD570 value was used for analysis. All absorbance readings were confirmed to fall within the linear detection range of the microplate reader. Samples exceeding the linear range were diluted appropriately and re-measured to prevent signal saturation and ensure accurate quantification.

#### 2.8.4. Classification of Biofilm Production

Wells containing sterile medium served as negative controls. Biofilm production was classified based on the mean OD570 of the three replicate wells relative to the negative control. Isolates with OD570 values comparable to the negative control were classified as non-biofilm producers, whereas progressively higher mean OD570 values were classified as weak, moderate, or strong biofilm producers, reflecting increasing biofilm biomass.

### 2.9. Data Analysis

The prevalence and distribution of ESBL-producing *K. pneumoniae* isolates were determined for each sampling site and sample type. Proportions of ESBL-positive isolates were calculated and summarized using descriptive statistics. PCR results were recorded as the presence or absence of targeted virulence genes. Biofilm assay data was analyzed by measuring optical density values obtained from the microtiter plate assay. Isolates were categorized as weak, moderate, or strong biofilm producers based on established cut-off values. Differences in biofilm-forming capacity between isolates from municipal wastewater and farm environments were evaluated using appropriate statistical tests, with significance set at *p* < 0.05. Statistical analyses were performed using R (v4.5.2) and Microsoft Excel.

## 3. Results

### 3.1. Prevalence of ESBL-Producing Klebsiella pneumoniae

A total of 449 environmental samples were collected and screened for the presence of ESBL-producing *K. pneumoniae* (Table 2). Overall, ESBL-producing *K. pneumoniae* was detected in 15.4% (69/449) of all samples analyzed. The distribution of positive isolates varied by environmental source. WWTPs accounted for the majority of ESBL-producing *K. pneumoniae* isolates, representing 75.4% (52/69) of all confirmed isolates, with 27 isolates recovered from WWTP-A and 25 from WWTP-B. Poultry farm samples contributed 21.7% (15/69) of the isolates, while the remaining isolates (2/69) were recovered from Cattle farm samples. Among wastewater-associated samples, influent (*n* = 31) and activated sludge (*n* = 12) samples yielded the highest number of ESBL-producing *K. pneumoniae* isolates.

### 3.2. Distribution of Virulence Genes

**Figure 1:**
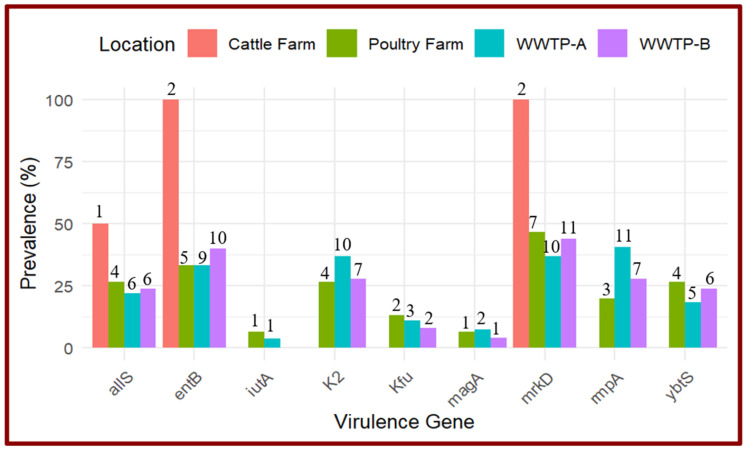
Distribution of virulence genes among ESBL-producing *Klebsiella pneumoniae* isolates by sampling location. Prevalence of selected virulence-associated genes among ESBL-producing *K. pneumoniae* isolates recovered from cattle farms, poultry farms, and wastewater treatment plants (WWTP-A and WWTP-B). Bars represent the percentage of isolates carrying each virulence gene within each sampling location. The most frequently detected genes across locations included *mrkD*, *entB*, *K2*, and *rmpA*. Numbers displayed on the bars indicate the number of isolates in each category.

PCR-based screening identified multiple virulence-associated genes among the ESBL-producing *K. pneumoniae* isolates. The most frequently detected virulence gene was *mrkD*, identified in 43.5% (30/69) of isolates, followed by *entB* in 37.7% (26/69), while both *K2* and *rmpA* were detected in 30.4% (21/69) of isolates. Virulence gene distribution varied across sampling locations (Figure 1). Among cattle farm isolates, *entB* and *mrkD* were each detected in 100% (2/2) of isolates, while *allS* was identified in 50.0% (1/2). In poultry farm isolates, *mrkD* was the most prevalent virulence gene, detected in 46.7% (7/15) of isolates, followed by *entB* in 33.3% (5/15). The genes *K2*, *allS*, and *ybtS* were each detected in 26.7% (4/15) of isolates, while *rmpA*, *Kfu*, *iutA*, and *magA* were detected at lower frequencies.

Among isolates recovered from WWTP-A, *rmpA* was the most common virulence gene, detected in 40.7% (11/27) of isolates, followed by *K2* and *mrkD* in 37.0% (10/27) each. In WWTP-B isolates, *mrkD* was the most frequently detected gene, identified in 44.0% (11/25) of isolates, followed by *entB* in 40.0% (10/25). Overall, *mrkD*, *entB*, *K2*, and *rmpA* were the predominant virulence-associated genes across wastewater and agricultural environments.

### 3.3. Virulence Gene Distribution by Sample Type and Location

**Figure 2:**
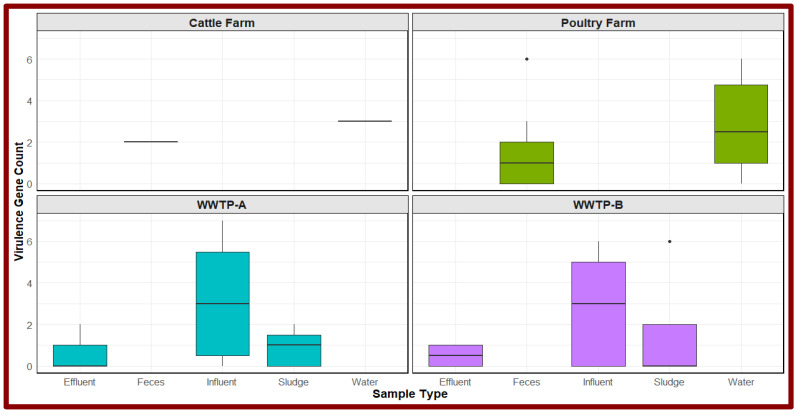
Virulence gene count among *K. pneumoniae* isolates by sample type and location. Virulence gene counts among ESBL-producing *K. pneumoniae* isolates stratified by sample type (influent, effluent, sludge, water, and feces) and location (WWTP-A, WWTP-B, poultry farm, and cattle farm). Bars represent the number of isolates positive for each virulence gene within each sample type and location. Individual dots indicate outlier observations.

Virulence gene distribution among ESBL-producing *K. pneumoniae* varied across sample types and locations (Figure 2). In wastewater treatment plants, influent samples harbored the highest diversity and frequency of virulence genes, with *mrkD*, *entB*, and *rmpA* being the most prevalent genes in both WWTP-A and WWTP-B. Sludge samples also contained multiple virulence determinants, although at lower frequencies compared to influent, with *K2*, *rmpA*, and *ybtS* commonly detected.

Effluent samples showed comparatively lower gene prevalence, with only a limited number of virulence genes detected across WWTP-A and WWTP-B, suggesting reduced bacterial load following treatment processes. However, the presence of *K2*, *rmpA*, and *ybtS* in effluent samples indicates incomplete removal of potentially pathogenic strains.

In agricultural environments, cattle farm water samples exhibited limited diversity, with *entB* and *mrkD* detected in all isolates (100%, 2/2), while poultry farm samples showed a broader distribution of virulence genes. In poultry farm water, *mrkD* (66.7%) and *entB* (50.0%) were the most frequent, followed by *K2*, *Kfu*, *allS*, *rmpA*, and *ybtS*, highlighting the presence of multiple virulence-associated traits in farm-associated isolates.

The distribution of virulence genes varied significantly according to sample source. Statistical analysis demonstrated significant differences in the occurrence of *mrkD* (*p* = 0.0013) and *entB* (*p* = 0.0011) among sample types. These findings indicate that certain environmental niches may favor the persistence or dissemination of specific virulence determinants.

The detection of genes associated with adhesion, iron acquisition, capsule production, and hypervirulence highlights the pathogenic potential of ESBL-producing *K. pneumoniae* isolates circulating within wastewater and agricultural environments.

### 3.4. Biofilm-Forming Capacity of ESBL-Producing Klebsiella pneumoniae Isolates

**Figure 3:**
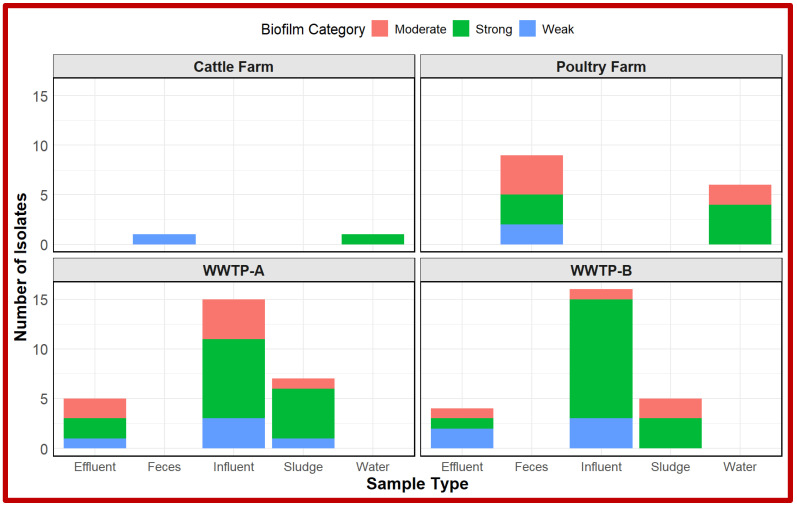
Distribution of biofilm-forming ESBL-producing *K. pneumoniae* isolates by sample type and location. Distribution of weak, moderate, and strong biofilm-forming *K. pneumoniae* isolates recovered from different sample types and locations, including poultry farms, cattle farms, and WWTPs. Biofilm formation was categorized based on the intensity of biofilm production measured using the microtiter plate assay. The figure illustrates variations in biofilm-forming capacity across environmental and farm-associated isolates.

Biofilm formation assays revealed variability in the ability of ESBL-producing *K. pneumoniae* isolates to produce biofilms. Across all sampled environments, isolates exhibiting Strong biofilm formation were the most frequently detected phenotype (56.5%; 39/69), followed by Moderate (24.6%; 17/69) and Weak (18.8%; 13/69). Biofilm strength varied markedly by location and sample type (Figure 3). Agricultural sites showed comparatively lower biofilm burdens: the cattle farm yielded only two isolates (one Weak in feces and one Strong in water), while the poultry farm (*n* = 15) displayed a broader distribution, including Strong biofilm producers in both feces and water (7/15), followed by Moderate (6/15) and Weak producers (2/15). In contrast, wastewater treatment plants (WWTPs) exhibited substantially higher counts of Strong biofilm producers. At WWTP-A, Strong biofilm isolates were detected in effluent (*n* = 2), influent (*n* = 8), and sludge (*n* = 5), with influent samples showing the highest concentration. WWTP-B demonstrated the greatest overall biofilm intensity, particularly in influent samples, where Strong biofilm producers reached a count of 16—the highest observed in the dataset. Moderate and Weak biofilm phenotypes were also present across WWTP sample types but at lower frequencies.

Although differences in biofilm-forming capacity were noted between environmental sources, statistical analysis showed no significant association between sample type and biofilm formation category (*p* = 0.357). Nevertheless, the predominance of strong biofilm producers in wastewater-associated samples suggests that these environments may support the persistence and survival of ESBL-producing *K. pneumoniae* through biofilm-mediated protection.

### 3.5. Phylogenetic Analysis and Genetic Relatedness

**Figure 4:**
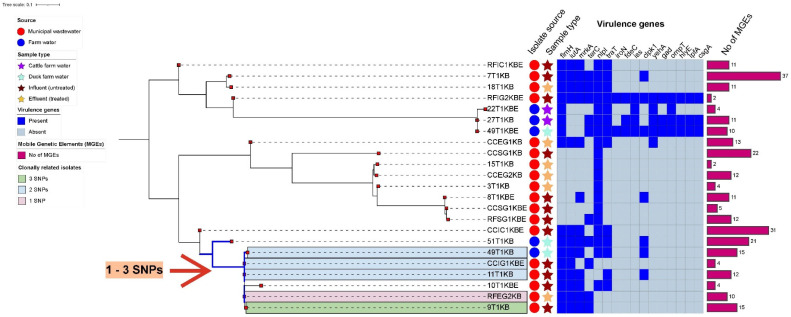
Phylogenetic relationship of *K. pneumoniae* isolates from agricultural and wastewater sources showing isolate source, sample type, virulence genes, mobile genetic elements (MGEs), and clonal relatedness. Maximum-likelihood phylogenetic tree of *K. pneumoniae* isolates recovered from poultry farm and municipal wastewater samples. Metadata displayed alongside the tree include isolate source, sample type, and the number of detected MGEs. A cluster of clonally related isolates differing by 1–3 single nucleotide polymorphisms (SNPs) was identified between one poultry isolate and four wastewater isolates, suggesting close genetic relatedness and possible dissemination across animal and environmental sources. Red squares indicate branch nodes. The arrow highlights a cluster of closely related isolates differing by 1–3 SNPs. Solid lines represent the main phylogenetic branches, while dashed lines connect terminal branches to isolate names to facilitate identification and improve readability.

All sequenced isolates were verified as *Klebsiella pneumoniae.* Phylogenetic reconstruction identified close clustering between several wastewater and poultry farm *K. pneumoniae* isolates, indicating potential environmental dissemination across interconnected systems. Notably, two poultry farm isolates were found to cluster with wastewater isolates on the phylogenetic tree (Figure 4). However, only one poultry isolate was closely related to four wastewater isolates, differing by only 1–3 SNPs. These isolates also shared key virulence-associated genes, including *mrkD*, *iutA*, and *fimH*. The high degree of genomic similarity observed among these isolates suggests possible transmission or common environmental sources linking wastewater and agricultural settings.

Several mobile genetic elements (MGEs), including insertion sequences (ISs), transposons, and miniature inverted-repeat transposable elements (MITEs), were identified among isolates from different sources (Figure 3). The highest MGE counts were observed in wastewater isolates, with up to 37 and 31 MGEs detected per isolate. These wastewater isolates were predominantly characterized by insertion sequences (*n* = 31) and transposons (*n* = 6). In comparison, farm-associated isolates harbored lower MGE counts, with a maximum of 21 MGEs comprising insertion sequences (*n* = 19), transposons (*n* = 1), and miniature inverted-repeat elements (*n* = 1). IS903 and IS26 were the most frequently identified insertion sequences among farm and wastewater isolates. The most commonly detected miniature inverted-repeat element was MITEEc-1, while the identified transposons occurred as singletons.

### 3.6. Association Between MGEs and Virulence Gene Profiles in K. pneumoniae Isolates

Wastewater and farm-associated *K. pneumoniae* isolates with the highest MGE counts were frequently associated with the presence of virulence genes including *fimH*, *iutA*, *mrkA*, *terC*, *nlpI*, *traT*, and *clpk1* (Figure 4). These isolates predominantly carried insertion sequences and transposons, suggesting a potential role of MGEs in the acquisition and dissemination of virulence genes. Notably, isolates recovered from wastewater exhibited the greatest MGE burden overall. In contrast, some isolates harboring relatively fewer MGEs possessed a broader repertoire of virulence genes, including *iroN*, *fdeC*, *iss*, *yehA*, *gad*, *ompT*, *hlyE*, *lpfA*, and *csgA*. These findings indicate variability in the relationship between MGE abundance and virulence gene carriage among the isolates.

## 4. Discussion

This study investigated the prevalence, virulence profiles, biofilm-forming capacity, and genomic relatedness of ESBL-producing *K. pneumoniae* isolated from WWTPs and agricultural environments. The findings demonstrate that wastewater systems, particularly influent and sludge samples, serve as important environmental reservoirs for virulent and multidrug-resistant *K. pneumoniae*. The high prevalence of ESBL-producing *K. pneumoniae* observed in WWTPs compared to animal farm environments highlights the role of wastewater systems in the accumulation and dissemination of AMR bacteria. The high recovery of ESBL-producing *K. pneumoniae* from influent and activated sludge in the present study indicates that wastewater systems may function as important environmental reservoirs for multidrug-resistant strains.

The predominance of virulence-associated genes such as *mrkD*, *entB*, *K2*, and *rmpA* among isolates in the present study suggests that environmental ESBL-producing *K. pneumoniae* strains possess important pathogenic traits linked to adhesion, iron acquisition, capsule formation, and hypervirulence [2,20,37]. The high frequency of *mrkD*, particularly among wastewater and poultry farm isolates, is notable because this gene is associated with type 3 fimbriae and biofilm formation, which enhance bacterial attachment and persistence on environmental surfaces [14,38,39,40]. Similarly, the presence of *entB* reflects the ability of these isolates to acquire iron in nutrient-limited environments, potentially increasing survival and competitiveness [23,41,42,43].

Our results show significant variation in the distribution of *mrkD* and *entB* across sample types. This indicates that environmental conditions may influence the selection and persistence of specific virulence determinants [14,42,43,44]. Wastewater environments are characterized by high microbial density, nutrient availability, and antimicrobial exposure, which may favor the maintenance of virulence and resistance-associated genes [1,17,19]. The detection of *K2* and *rmpA*, genes commonly associated with hypervirulent *K. pneumoniae* strains, further raises concerns regarding the pathogenic potential of isolates circulating in environmental reservoirs [2,23,43,45]. Similar findings have been reported in environmental and clinical studies, where wastewater systems were identified as reservoirs of multidrug-resistant and virulent Enterobacterales, often enriched with adhesion and iron-scavenging genes due to high microbial density and selective pressure from antimicrobial residues [8,20,23,41,42,43,46].

Our results showed the detection of virulence genes in effluent samples, although at lower frequencies. This indicates incomplete removal of bacteria carrying virulence-associated genes during wastewater treatment, consistent with previous reports showing persistence of ESBL-producing Enterobacterales in treated effluents [47,48,49]. This highlights the potential for environmental dissemination into receiving water bodies. In agricultural settings, the higher diversity of virulence genes observed in poultry farm isolates compared to cattle farm isolates aligns with previous studies that have identified poultry production systems as important hotspots for AMR *Klebsiella* due to dense animal populations and frequent antimicrobial exposure [50,51].

Biofilm formation assays in the present study demonstrated that strong biofilm production was most frequently observed among influent and sludge isolates. Although no statistically significant differences in biofilm formation were identified across sample types, the predominance of strong biofilm producers in wastewater-associated samples suggests that wastewater systems provide favorable conditions for biofilm development [19,22,23,24,52]. Biofilm formation is an important survival strategy that enhances bacterial persistence, protects organisms from environmental stressors and disinfection processes, and facilitates horizontal gene transfer, including the dissemination of antimicrobial resistance genes [23,52,53].

Phylogenetic analysis revealed close genetic relatedness between poultry farm and wastewater isolates, with some isolates differing by only 1–3 SNPs. The shared presence of virulence genes such as *mrkD*, *iutA*, and *fimH* among clonally related isolates suggests possible environmental dissemination or transmission between wastewater and agricultural settings [11,53,54]. These findings support the hypothesis that wastewater systems may contribute to the movement of resistant and potentially pathogenic *K. pneumoniae* strains into agricultural environments through contaminated water, runoff, or sludge application [19,55,56]. The high degree of genomic similarity observed among these isolates suggests possible transmission or common environmental sources linking wastewater and agricultural settings [11,53].

Our results showed high diversity and abundance of MGEs identified among the *K. pneumoniae* isolates, particularly those recovered from wastewater. This may contribute significantly to the dissemination of virulence-associated traits and enhanced biofilm formation [14,57,58]. Wastewater isolates in the present study harbored the greatest number of MGEs, dominated by insertion sequences and transposons, suggesting increased genomic plasticity and an elevated capacity for the acquisition and spread of virulence determinants [57,58,59,60]. Insertion sequences such as IS903 have been associated with genomic rearrangements and regulation of genes involved in bacterial adaptation, persistence, and pathogenicity [61]. The presence of these elements in isolates with multiple virulence genes may facilitate the mobilization and expression of factors involved in adhesion, colonization, iron acquisition, and biofilm production [57,58,59,60]. The comparatively lower MGE burden observed among farm isolates observed in our study may reflect reduced selective pressures; however, the detection of insertion sequences and MITEEc-1 indicates ongoing genomic evolution and the potential for virulence gene dissemination within these populations [57,59,60,62]. The predominance of IS26 among the isolates in the present study is noteworthy, as IS26 are clinically important insertion sequences known to facilitate genomic rearrangements and the mobilization of virulence and antimicrobial resistance genes, thereby contributing to bacterial adaptation and persistence across diverse environments [63].

The association between high MGE counts and virulence genes such as *fimH*, *iutA*, *mrkA*, *terC*, *nlpI*, *traT*, and *clpK1* suggests that MGEs may contribute to the persistence, adaptation, and pathogenic potential of *K. pneumoniae* in both wastewater and farm environments [58]. The increased abundance of MGEs in wastewater isolates further supports the role of wastewater systems as hotspots for horizontal gene transfer and genetic exchange [57,58,59,60]. However, the observation that some isolates with fewer MGEs carried a larger number of virulence genes highlights that virulence potential is not solely dependent on MGE abundance. These virulence-rich isolates possessed genes such as *iroN*, *iss*, *ompT*, *hlyE*, and *csgA*, which are associated with serum resistance, toxin production, adhesion, and biofilm formation, suggesting that stable chromosomal integration or previously acquired virulence determinants may also contribute substantially to pathogenicity [2,12,14,20,23].

Overall, the presence of closely associated virulence determinants across wastewater and farm environments supports the hypothesis of environmental connectivity and potential cross-transmission [11,53,54]. These findings reinforce the need for integrated One Health surveillance strategies targeting wastewater and agricultural interfaces to limit the spread of virulent and AMR *Klebsiella* spp. [4,5,38,64]. Improved wastewater management, environmental surveillance, and biosecurity practices are needed to reduce the dissemination of ESBL-producing pathogens and mitigate risks to environmental, animal, and human health [1,6,64,65].

The study has several important limitations that should be considered when interpreting the findings. Only a small number of farms and wastewater treatment plants were included, which narrows the extent to which the results can be applied to other regions or production systems. Sampling intensity and the availability of specific matrices also differed among sites due to logistical and access challenges encountered during fieldwork, introducing uneven representation across locations. Because sampling occurred exclusively between May and September 2025, the observed ESBL-producing *K. pneumoniae* prevalence likely reflects late spring and summer conditions; year-round monitoring would be required to evaluate potential seasonal trends. Financial constraints further limited the number of isolates subjected to sequencing, reducing the depth of genomic characterization across the full isolate set. Consequently, species identification of the non-sequenced isolates could not be confirmed genomically and should be interpreted with caution. Sequenced isolates were chosen based on virulence gene content and source category, which may influence how well the genomic subset reflects the broader collection. Despite these constraints, the study contributes important evidence on the virulence traits, biofilm-forming capacity, and genomic characteristics of ESBL-producing *K. pneumoniae* circulating in agricultural and environmental settings, and it establishes a basis for future investigations with broader scope.

## 5. Conclusions

This study illustrates that ESBL-producing *K. pneumoniae* is both widespread and genetically diverse across agricultural and environmental reservoirs, reinforcing the interconnected nature of farms and wastewater systems in shaping the distribution of virulence traits, biofilm capacity, and mobile genetic elements. Evidence of clonal relatedness between isolates from poultry and municipal wastewater highlights the potential for possible movement between reservoirs and underscores the need to consider these environments as part of a shared transmission landscape. By characterizing virulence profiles, biofilm formation, and genomic features across multiple sources, the study advances understanding of the ecological and genetic drivers that facilitate ESBL-producing *K. pneumoniae* dissemination. Strengthening integrated One Health-based surveillance across animal farms, municipal wastewater, and environmental compartments will be essential for mitigating the spread of hypervirulent *K. pneumoniae* strains. Future work should broaden sampling coverage and expand whole-genome sequencing efforts to improve representativeness, resolve transmission pathways, and inform targeted interventions aimed at reducing the emergence and environmental release of resistant bacteria.

## Figures and Tables

**Table 1:** Primers used for multiplex PCR detection of virulence-associated genes in *Klebsiella pneumoniae* isolates.

Virulence Genes	Function	Forward—Rimers DNA Sequence (5′ to 3′)	Reverse—Primers DNA Sequence (5′ to 3′)	Amplicon Sizes (bp)	References
Multiplex PCR Assay A					
*mrkD*	Adhesin type 3 fimbriae	AAGCTATCGCTGTACTTCCGGCA	GGCGTTGGCGCTCAGATAGG	340	Compain et al. (2014) [26]
*entB*	Siderophore	GTCAACTGGGCCTTTGAGCCGTC	TATGGGCGTAAACGCCGGTGAT	400	
*allS*	Allantoin metabolism	CATTACGCACCTTTGTCAGC	GAATGTGTCGGCGATCAGCTT	764	
*iutA*	Siderophore	GGGAAAGGCTTCTCTGCCAT	TTATTCGCCACCACGCTCTT	920	
*Kfu*	Iron transport and phosphotransferase function	GGCCTTTGTCCAGAGCTACG	GGGTCTGGCGCAGAGTATGC	638	
Multiplex PCR assay B					
*ybtS*	Siderophore	GACGGAAACAGCACGGTAAA	GAGCATAATAAGGCGAAAGA	242	Compain et al. (2014) [26]
*rmpA*	Regulator of mucoid phenotype A	CATAAGAGTATTGGTTGACAG	CTTGCATGAGCCATCTTTCA	461	
*magA*	Capsular serotype and hypermucoviscosity phenotype	GGTGCTCTTTACATCATTGC	GCAATGGCCATTTGCGTTAG	1283	
*K2*		CAACCATGGTGGTCGATTAG	TGGTAGCCATATCCCTTTGG	531	

**Table 2:** Sampling framework and recovery of ESBL-producing *K. pneumoniae* from wastewater and farm environments.

Sample Type	Samples Collected *n* (%) *n* = 449	*Klebsiella* Positive *n* (%) *n* = 141	ESBL-*Klebsiella* Positive *n* (%) *n* = 69	Sequenced Isolates *n* (%) *n* = 27
Wastewater (*n* = 166)				
Effluent	94 (20.9)	36 (25.5)	9 (13.0)	7 (25.9)
Influent	48 (10.7)	36 (25.5)	30 (43.5)	6 (22.2)
Sludge	24 (5.3)	18 (12.8)	13 (18.8)	6 (22.2)
Cattle Farm (*n* = 126)				
Feces	30 (6.7)	1 (0.7)	1 (1.4)	1 (3.7)
Water	66 (14.7)	5 (3.5)	1 (1.4)	4 (14.8)
Soil	30 (6.7)	3 (2.1)	0 (0.0)	0 (0.0)
Poultry Farm (*n* = 157)				
Chicken feces	58 (12.9)	6 (4.3)	3 (4.3)	0 (0.0)
Duck feces	42 (9.4)	5 (3.5)	3 (4.3)	0 (0.0)
Chicken water	24 (5.3)	20 (14.2)	6 (8.7)	0 (0.0)
Duck water	33 (7.3)	11 (7.8)	3 (4.3)	3 (11.1)

## Data Availability

Data generated and analyzed during this study are included within the article. Additional information may be obtained from the corresponding authors upon reasonable request.

## Supplementary Materials

The following supporting information can be downloaded at: https://www.mdpi.com/article/10.3390/microorganisms14071435/s1, Table S1: Metadata for the *K. pneumoniae* isolates included in this study, including isolate name, source, country, collection year, and accession numbers.

## References

[1] Hu Y., Anes J., Devineau S., Fanning S. (2021). *Klebsiella pneumoniae*: Prevalence, Reservoirs, Antimicrobial Resistance, Pathogenicity, and Infection: A Hitherto Unrecognized Zoonotic Bacterium. Foodborne Pathog. Dis..

[2] Riwu K.H.P., Effendi M.H., Rantam F.A., Khairullah A.R., Widodo A. (2022). A review: Virulence factors of *Klebsiella pneumonia* as emerging infection on the food chain. Vet. World.

[3] Abbas R., Chakkour M., Zein H., Dine E., Obaseki E.F., Obeid S.T. (2024). General Overview of *Klebsiella pneumonia*: Epidemiology and the Role of Siderophores in Its Pathogenicity. Biology.

[4] Ramirez-Plascencia H.H.F., Colima-Fausto A.G., Licona-Lasteros K.C., Díaz-Zaragoza M., Cazarez-Navarro G., Macias-Barragan J.G., Rodriguez-Preciado S.Y. (2025). Presence of Microorganisms in the Environment: One Health Approach. Microorganisms.

[5] Wall K., Macori G., Koolman L., Li F., Fanning S. (2023). Klebsiella, a Hitherto Underappreciated Zoonotic Pathogen of Importance to One Health: A Short Review. Zoonoses.

[6] Microbiol C., Effah C.Y., Sun T., Liu S., Wu Y. (2020). *Klebsiella pneumoniae*: An increasing threat to public health. Ann. Clin. Microbiol. Antimicrob..

[7] Woh P.Y., Zhang X. (2025). The burden of ESKAPE pathogen-related hospital-acquired infections: Clinical and financial perspective from a systematic review. J. Hosp. Infect..

[8] Huang X., Yao X., Hou Y., Zhang D., Xie R., Shi C., Shang Y., Bi H., Song W., Hua L. (2025). Global trends of antimicrobial resistance and virulence of *Klebsiella pneumoniae* from different host sources. Commun. Med..

[9] Song H., Lin Y., Wan T., Yang G., Jiang M., Fu B., Pan J.-S. (2025). Global burden of *Klebsiella pneumoniae* infections and antimicrobial resistance in 2019. BMC Infect. Dis..

[10] World Health Organization (WHO) (2024). WHO Bacterial Priority Pathogens List, 2024: Bacterial Pathogens of Public Health Importance to Guide Research, Development and Strategies to Prevent and Control Antimicrobial Resistance.

[11] Maldonado K.V.M., Pereira J.M., Costa T.N.D., Buss G.L., Almeida Pereira K.N.D., Silva A.B.D., Corção G., Ândrea de Souza C., Martins A.S., Falci D.R. (2025). Widespread Distribution of Carbapenem-Resistant Klebsiella spp. in Clinical and Environmental Settings. Antibiotics.

[12] Araújo S., Silva V., Quintelas M., Martins Â., Igrejas G., Poeta P. (2025). From soil to surface water: Exploring Klebsiella ’s clonal lineages and antibiotic resistance odyssey in environmental health. BMC Microbiol..

[13] Chawla K., Piveteau P., Sharma S. (2025). *Klebsiella pneumoniae*: A connecting link in the One Health concept. Pathog. Glob. Health.

[14] Rahman H., Akther S., Ahmed S., Shahadat N., Munsi N. (2025). Epidemiological factors associated with the prevalence of mobile genetic elements, and antimicrobial resistance patterns in *Klebsiella pneumoniae* of farm environments in Bangladesh. BMC Microbiol..

[15] Burch T.R., Newton R.J., Kimbell L.K., Marshall C.W., Mcnamara P.J., Lou Lamartina E. (2022). Targeting current and future threats: Recent methodological trends in environmental antimicrobial resistance research and their relationships to risk assessment. Environ. Sci. Water Res. Technol..

[16] Navon-venezia S., Kondratyeva K., Carattoli A. (2017). *Klebsiella pneumoniae*: A major worldwide source and shuttle for antibiotic resistance. FEMS Microbiol. Rev..

[17] Le L.T., Huang Z., Whiteson K., Jiang S. (2022). The occurrence and diversity of antibiotic resistance and virulence factor genes in wastewater from four North American treatment plants. Environ. Sci. Water Res. Technol..

[18] Loudermilk E.M., Kotay S.M., Barry K.E., Parikh H.I., Colosi L.M., Mathers A.J. (2022). Tracking *Klebsiella pneumoniae* carbapenemase gene as an indicator of antimicrobial resistance dissemination from a hospital to surface water via a municipal wastewater treatment plant. Water Res..

[19] Marutescu L.G., Popa M., Gheorghe-Barbu I., Barbu I.C., Rodríguez-Molina D., Berglund F., Blaak H., Flach C.-F., Kemper M.A., Spießberger B. (2023). Wastewater treatment plants, an “escape gate” for ESCAPE pathogens. Front. Microbiol..

[20] Rolbiecki D., Harnisz M., Korzeniewska E., Buta M., Hubeny J., Zieliński W. (2021). Detection of carbapenemase-producing, hypervirulent Klebsiella spp. in wastewater and their potential transmission to river water and WWTP employees. Int. J. Hyg. Environ. Health.

[21] Li Y., Ni M. (2023). Regulation of biofilm formation in *Klebsiella pneumoniae*. Front. Microbiol..

[22] Ashwath P., Deekshit V.K., Rohit A., Dhinakaran I., Karunasagar I., Karunasagar I., Akhila D.S. (2022). Biofilm Formation and Associated Gene Expression in Multidrug-Resistant *Klebsiella pneumoniae* Isolated from Clinical Specimens. Curr. Microbiol..

[23] Ballén V., Gabasa Y., Ratia C., Ortega R., Tejero M., Soto S. (2021). Antibiotic Resistance and Virulence Profiles of *Klebsiella pneumoniae* Strains Isolated From Different Clinical Sources. Front. Cell Infect. Microbiol..

[24] Lordelo R., Branco R., Gama F., Morais P.V. (2024). Assessment of antimicrobial resistance, biofilm formation, and surface modification potential in hospital strains of *Pseudomonas aeruginosa* and *Klebsiella pneumoniae*. Heliyon.

[25] Appling K.C., Sobsey M.D., Durso L.M., Fisher M.B. (2023). Environmental monitoring of antimicrobial resistant bacteria in North Carolina water and wastewater using the WHO Tricycle protocol in combination with membrane filtration and compartment bag test methods for detecting and quantifying ESBL *E. coli*. PLoS Water.

[26] Compain F., Babosan A., Brisse S., Genel N., Audo J., Ailloud F. (2014). Multiplex PCR for Detection of Seven Virulence Factors and K1/K2 Capsular Serotypes of *Klebsiella pneumoniae*. J. Clin. Microbiol..

[27] Timme R., Shrestha Y., Pfefer T., Morin P., Balkey M., Strain E. (2023). Quality Control Assessment for Microbial Genomes: GalaxyTrakr MicroRunQC Workflow.

[28] Wick R.R., Judd L.M., Gorrie C.L., Holt K.E. (2017). Unicycler: Resolving bacterial genome assemblies from short and long sequencing reads. PLoS Comput. Biol..

[29] Seemann T. (2014). Prokka: Rapid prokaryotic genome annotation. Bioinformatics.

[30] Tonkin-Hill G., MacAlasdair N., Ruis C., Weimann A., Horesh G., Lees J.A., Gladstone R.A., Lo S., Beaudoin C., Floto R.A. (2020). Producing polished prokaryotic pangenomes with the Panaroo pipeline. Genome Biol..

[31] Page A.J., Taylor B., Delaney A.J., Soares J., Seemann T., Keane J.A., Harris S.R. (2016). SNP-sites: Rapid efficient extraction of SNPs from multi-FASTA alignments. Microb. Genom..

[32] Seemann T., Klotzl F., Page A. (2018). SNP-dists: Pairwise SNP Distance Matrix from a FASTA Sequence Alignment.

[33] Kozlov A.M., Darriba D., Flouri T., Morel B., Stamatakis A. (2019). RAxML-NG: A fast, scalable and user-friendly tool for maximum likelihood phylogenetic inference. Bioinformatics.

[34] Letunic I., Bork P. (2024). Interactive Tree of Life (iTOL) v6: Recent updates to the phylogenetic tree display and annotation tool. Nucleic Acids Res..

[35] Johansson M.H.K., Bortolaia V., Tansirichaiya S., Aarestrup F.M., Roberts A.P., Petersen T.N. (2021). Detection of mobile genetic elements associated with antibiotic resistance in Salmonella enterica using a newly developed web tool: MobileElementFinder. J. Antimicrob. Chemother..

[36] Naves P., del Prado G., Huelves L., Gracia M., Ruiz V., Blanco J., Rodríguez-Cerrato V., Ponte M., Soriano F. (2008). Measurement of biofilm formation by clinical isolates of Escherichia coli is method-dependent. J. Appl. Microbiol..

[37] Ahmadi M., Ranjbar R., Behzadi P., Mohammadian T. (2022). Virulence factors, antibiotic resistance patterns, and molecular types of clinical isolates of *Klebsiella pneumoniae*. Expert. Rev. Anti Infect. Ther..

[38] Deepan G., Rekha V.B., Kumar V.J.A. (2022). Type 1 and 3 Fimbriae as Key Factors in Biofilm Development of *Klebsiella pneumoniae*: A One Health Perspective in the Context of Food Safety. J. Vet. Public Health.

[39] Ahmed Z.S., Abdel-kader F., Hamza D., Ashour R. (2026). Molecular detection of hypervirulent *Klebsiella pneumoniae* (hvKp) in Egyptian poultry. BMC Vet. Res..

[40] Khalefa H.S., Arafa A.A., Hamza D., El-razik K.A.A., Ahmed Z. (2025). Emerging biofilm formation and disinfectant susceptibility of ESBL-producing *Klebsiella pneumoniae*. Sci. Rep..

[41] Garc I., Kürekci C., Ünaldı Ö., Sahin S. (2024). Impact and Diversity of ESBL-Producing *Klebsiella pneumoniae* Recovered from Raw Chicken Meat Samples in Türkiye. Antibiotics.

[42] Lan P., Lu Y., Fu Y., Yu Y., Zhou J. (2025). Siderophores and beyond: A comprehensive review of iron acquisition in *Klebsiella pneumoniae*. Virulence.

[43] Zhu J., Wang T., Chen L., Du H. (2021). Virulence Factors in Hypervirulent *Klebsiella pneumoniae*. Front. Microbiol..

[44] Mandujano-Hernández A., Martínez-Vázquez A.V., Paz-González A.D., Herrera-Mayorga V., Sánchez-Sánchez M., Lara-Ramírez E.E., Vázquez K., de Jesús de Luna-Santillana E., Bocanegra-García V., Rivera G. (2024). The Global Rise of ESBL-Producing Escherichia coli in the Livestock Sector: A Five-Year Overview. Animals.

[45] Al Ismail D., Campos-Madueno E.I., Donà V., Endimiani A. (2025). Hypervirulent *Klebsiella pneumoniae* (hvKp): Overview, Epidemiology, and Laboratory Detection. Pathog. Immun..

[46] Thirugnanasambantham M.K., Thuthikkadu Indhuprakash S., Thirumalai D. (2025). Phenotypic and Genotypic Characteristics of Hypervirulent *Klebsiella pneumoniae* (hvKp): A Narrative Review. Curr. Microbiol..

[47] Fadare F.T., Okoh A.I. (2021). The Abundance of Genes Encoding ESBL, pAmpC and Non-β-Lactam Resistance in Multidrug-Resistant Enterobacteriaceae Recovered From Wastewater Effluents. Front. Environ. Sci..

[48] Hasani K., Sadeghi H., Vosoughi M., Sardari M., Manouchehrifar M. (2023). Characterization of beta-lactamase producing Enterobacterales isolated from an urban community wastewater treatment plant in Iran. Iran. J. Microbiol..

[49] Puljko A., Babić I., Dekić S., Barišić I., Jelić M., Maravić A. (2024). Treated municipal wastewater as a source of high-risk and emerging multidrug-resistant clones of *E. coli* and other Enterobacterales producing extended-spectrum β-lactamases. Environ. Res..

[50] Hou G., Ahmad S., Li Y., Yan D., Yang S., Chen S., Qiu Z., Yu X., Li N., Li Y. (2024). Epidemiological, Virulence, and Antibiotic Resistance Analysis of *Klebsiella pneumoniae*, a Major Source of Threat to Livestock and Poultry in Some Regions of Xinjiang, China. Animals.

[51] Xiao X., Yang P., Peng K., Li Y., Wang Q., Lv Y., Feil E., Wang Z., Li R. (2025). Genomic Diversity and Coexistence of Multidrug-Resistance Mechanisms of *Klebsiella pneumoniae* in Poultry Farms. Transbound. Emerg. Dis..

[52] Li D., Jarocki V.M., Wyrsch E.R., Cummins M.L., Djordjevic S.P. (2025). Next generation ESKAPE-E superbugs: Identifying transmissible locus of stress tolerance and antibiotic resistance in pandemic bacterial lineages. npj Antimicrob. Resist..

[53] Zhang S., Yang J., Abbas M., Yang Q., Li Q., Liu M., Zhu D., Wang M., Tian B., Cheng A. (2025). Threats across boundaries: The spread of ESBL-positive Enterobacteriaceae bacteria and its challenge to the “one health” concept. Front. Microbiol..

[54] Pinamonti D., Vidic J., Maifreni M., Cossettini A., Leguillier V., Manzano M. (2025). Water-Mediated Dissemination and Detection of Antibiotic Resistance Across Livestock, Agri-Food, and Aquaculture Systems. Micromachines.

[55] Mabeo O.R., Van Niekerk B., Olanrewaju O.S., Bezuidenhout C.C., Molale-tom L.G. (2025). Comprehensive genome analysis of MDR *Klebsiella pneumoniae* in influent and effluent of a selected wastewater treatment plant. Sci. Rep..

[56] Abdelgalel R.R., Ibrahem R.A., Ahmed A.B.F., Mohamed D.S. (2026). persistence in wastewater: Co-occurrence of virulence and multidrug resistance genes. BMC Microbiol..

[57] Han B., Feng C., Jiang Y., Ye C., Wei Y., Liu J., Zeng Z. (2025). Mobile genetic elements encoding antibiotic resistance genes and virulence genes in *Klebsiella pneumoniae*: Important pathways for the acquisition of virulence and resistance. Front. Microbiol..

[58] Li Y., Farzana R. (2026). Mobile genetic elements in shaping *Klebsiella pneumoniae* pathogenicity. Front. Microbiol..

[59] Hossain H., Ali H., Ahmad T., Sharmin S., Sayeed B., Sakib A.N., Brishty K.A., Saleh M.S.J., Hosen M.M., Ahmed S. (2026). Mobile Genetic Elements as Central Drivers of Antimicrobial Resistance: Molecular Mechanisms, Evolutionary Ecology, One Health Implications and Control Strategies. Antibiotics.

[60] Pan T., Li Q. (2025). Mobile genetic elements in *Klebsiella pneumoniae*. J. Bacteriol..

[61] Shankar C., Pragasam A.K., Anandan S., Veeraraghavan B. (2019). mgrB as Hotspot for Insertion Sequence Integration: Change Over from Multidrug-Resistant to Extensively Drug-Resistant *Klebsiella pneumoniae*?. Microb. Drug Resist..

[62] Wei D.-W., Song Y., Li Y., Zhang G., Chen Q., Wu L., Huang J., Tian X., Wang C., Feng J. (2025). Insertion sequences accelerate genomic convergence of multidrug resistance and hypervirulence in *Klebsiella pneumoniae* via capsular phase variation. Genome Med..

[63] Varani A., He S., Siguier P., Ross K., Chandler M. (2021). The IS 6 family, a clinically important group of insertion sequences including IS 26. Mob. DNA.

[64] Milenkov M., Proux C., Rasolofoarison T.L., Rakotomalala F.A., Rasoanandrasana S., Rahajamanana V.L., Rafalimanana C., Ravaoarisaina Z., Ramahatafandry I.T., Westeel E. (2024). Implementation of the WHO Tricycle protocol for surveillance of extended-spectrum β-lactamase producing Escherichia coli in humans, chickens, and the environment in Madagascar: A prospective genomic epidemiology study. Lancet Microbe.

[65] Liguori K., Keenum I., Davis B.C., Calarco J., Milligan E., Harwood V.J., Pruden A. (2022). Antimicrobial Resistance Monitoring of Water Environments: A Framework for Standardized Methods and Quality Control. Environ. Sci. Technol..

